# GRAMD1A Is a Biomarker of Kidney Renal Clear Cell Carcinoma and Is Associated with Immune Infiltration in the Tumour Microenvironment

**DOI:** 10.1155/2022/5939021

**Published:** 2022-07-11

**Authors:** Yifu Liu, Shengqiang Fu, Zhicheng Zhang, Siyuan Wang, Xiaofeng Cheng, Zhilong Li, Yi Ding, Ting Sun, Ming Ma

**Affiliations:** Department of Urology, The First Affiliated Hospital of Nanchang University, Nanchang, 330000 Jiangxi Province, China

## Abstract

**Background:**

GRAM structural domain-containing protein 1A (GRAMD1A) is upregulated in a variety of human cancer tissues and is closely associated with tumourigenesis and progression.

**Methods:**

Patient RNA-sequencing data and clinicopathological information were obtained from The Cancer Genome Atlas (TCGA) and Gene Expression Omnibus (GEO) datasets. The expression of GRAMD1A in kidney cancer cell lines and KIRC patients was analysed by quantitative polymerase chain reaction (qPCR). Receiver Operator Characteristic (ROC) curves, nomograms, Kaplan-Meier analysis, forest plots, and COX analysis were used to assess the diagnostic and prognostic value of GRAMD1A in KIRC, and gene set enrichment analysis (GSEA) was used to explore its potential signalling pathways. In addition, the Sangerbox website, Kaplan-Meier plotter database, and TISIDB and TIMER databases were used to further analyse the correlation of GRAMD1A with microsatellite instability (MSI), tumour mutational burden (TMB), immune checkpoint genes, and tumour-infiltrating lymphocytes (TILs).

**Results:**

GRAMD1A was significantly highly expressed in KIRC and associated with shorter overall survival and relapse-free survival (*P* < 0.05). The AUC value of the ROC curve to identify KIRC and normal renal tissues was 0.942. Forest plot and COX analysis visualized that GRAMD1A could be an independent prognostic factor in KIRC patients (*P* < 0.01), and nomograms to determine the overall survival (OS) of KIRC patients also showed good efficacy (C-index: 0.776). Moreover, Spearman correlation analysis showed a positive correlation between GRAMD1A and MSI, TMB (*P* < 0.01). On the other hand, GRAMD1A was also found to be closely associated with immune checkpoint genes. Meanwhile, patients with KIRC with high GRAMD1A expression had a relatively low hazard ratio (HR) of death when B lymphocytes, natural killer T cells, CD4+ T lymphocytes, CD8+ T lymphocytes, and macrophages were enriched in the tumour microenvironment (TME), and a greater HR of death when regulatory T lymphocytes with tumour-specific immunosuppressive effects were significantly enriched. Last, GSEA shows that GRAMD1A is closely associated with the regulation of energy metabolism in KIRC.

**Conclusions:**

GRAMD1A is a promising diagnostic and prognostic biomarker for patients with KIRC, and its biological function correlates to some extent with immune infiltration in TME.

## 1. Introduction

In 2020, the number of incidences and deaths from renal cell carcinoma (RCC) is 2% of all cancer cases in 185 countries worldwide, and the social burden of RCC will continue to grow over the next 20 years based on population growth and ageing [1]. Kidney renal clear cell carcinoma (KIRC), the most common pathological type of renal cell carcinoma, has undoubtedly become a global public health problem [2]. Although the systemic treatment of KIRC has made great progress in recent years, a satisfactory prognosis for patients with local recurrence and distant metastases after radical surgery is still difficult to achieve [3]. First-line treatment for advanced disease is currently focused on targeted, tumour angiogenesis and immune system [4–7]; however, the long-term efficacy of these therapies remains a significant challenge due to the lack of adequate therapeutic targets and relevant biomarkers, as well as a lack of understanding of the pathophysiological mechanisms underlying the development of KIRC.

GRAM domain-containing protein 1A (GRAMD1A), originally identified in human embryonic stem cells, is 724 amino acids in length and contains a GRAM structural domain [8]; it is thought to be an intracellular protein-binding or lipid-binding signalling domain and may play an important role in membrane-associated processes [9]. GRAMD1A is found to be expressed in a variety of human cancer tissues, including the digestive, urinary, and reproductive systems [8]. In addition, GRAMD1A was found to promote the expansion of hepatocellular carcinoma stem cells, the growth of hepatocellular carcinoma, and resistance to chemotherapy via STAT5 [10]. Meanwhile, upregulation of GRAMD1A expression promoted proliferation, migration, and invasion of hepatocellular carcinoma in in vitro assays [11].

However, to date, the potential role of GRAMD1A in patients with KIRC remains unclear. Thus, in this study, we first obtained RNA-sequencing data and clinical information from KIRC patients through The Cancer Genome Atlas (TCGA) and Gene Expression Omnibus (GEO) datasets and then found that GRAMD1A expression was upregulated in KIRC patients and associated with more aggressive clinicopathological features. Furthermore, we assessed the diagnostic and prognostic value of GRAMD1A in KIRC, comprehensively analysed the relationship between GRAMD1A and immunological features, and finally explored its possible signalling pathways and pathophysiological mechanisms through gene set enrichment analysis (GSEA).

## 2. Materials and Methods

### 2.1. Data Access

RNA-sequencing data from 539 KIRC tissues and 72 normal kidney tissues and clinicopathological information on patients were downloaded from the TCGA database (https://portal.gdc.cancer.gov/) [12]. Besides, we also obtained RNA-seq expression data for GRAMD1A in KIRC tissue and normal tissue from a number of GEO datasets (https://www.ncbi.nlm.nih.gov/gds/) [13], including GSE105261, GSE36895, and GSE53757.

### 2.2. Quantitative PCR (qPCR)

Total RNA was extracted using Trizol reagent (Cwbio, China) from cell lines (HK-2, A498, 769-P, 786-O, Caki-1, and OSRC-2) and paired tissues from 19 pairs of KIRC patients from our centre, followed by the cDNA synthesis kit (Qiagen, USA) and SYBR real-time PCR kit (Qiagen, USA) for reverse transcription and qPCR, respectively; the 2-*ΔΔ*Ct method was used for relative quantification. The primer sequences are as follows: GRAMD1A forward: GATGCTCTCTTCTCGGACTCG, reverse: GATGGGGATGGTGTACGTC; *β*-actin forward: TCTCCCAAGTCCACACAGG, reverse: GGCACGAAGGCTCATCA.

### 2.3. UALCAN and HPA Datasets

Expression analysis of GRAMD1A at the protein level was obtained from the UALCAN database (http://ualcan.path.uab.edu/) [14]. The HPA database (https://www.proteinatlas.org/) was used to show the expression of GRAMD1A in normal kidney tissue and KIRC [15].

### 2.4. Sangerbox Website Analysis

The Sangerbox website (http://www.sangerbox.com/tool) is a free data analysis platform that we have used to analyse the expression of GRAMD1A pan-cancer and to explore its association with microsatellite instability (MSI) and tumour mutational burden (TMB) [16]. And, we analysed the correlation between GRAMD1A and immune infiltrating lymphocytes in KIRC based on the CIBERSORT algorithm [17].

### 2.5. Kaplan-Meier Plotter Database Analysis

The Kaplan Meier plotter (http://kmplot.com/analysis/) is a publicly available tumour prognostic analysis tool and is capable of assessing the survival impact of tens of thousands of genes across 21 cancer types [18]. We used this tool to explore the impact of GRAMD1A on the survival prognosis of various cancers and also to compare the impact of GRAMD1A on the prognosis of KIRC patients when tumour-infiltrating lymphocytes (TILs) are enriched and reduced.

### 2.6. Construction and Evaluation of Prognostic Models

Nomogram models were constructed to help us visualize the effect of individual predictors (age, T, N, M, histological grade, and GRAMD1A expression level) on overall survival (OS) and also to help clinicians make judgments about the prognosis of patients. Time-dependent ROC curves and calibration curves were used to assess the predictive value and accuracy of the nomogram model. Both analyses were performed using the R “rms” package.

### 2.7. TISIDB and TIMER Database Analysis

TISIDB (http://cis.hku.hk/tisidb/) is a portal for tumour-immune system interactions, including 30 cancer types from the TCGA database [19], and TIMER (http://timer.cistrome.org/) is a comprehensive resource for systematical analysis of immune infiltrates across diverse cancer types [20]. Both tools are used to explore the correlation between GRAMD1A expression and immune checkpoint genes in KIRC.

### 2.8. GSEA Analysis

GSEA is used to reveal the potential biological pathway of GRAMD1A in KIRC, with at least 1000 operations per analysis, and results with *P* < 0.05 and false discovery rate (FDR) < 0.25 were considered significant [21].

### 2.9. Statistical Analyses

RNA-sequencing expression data as well as clinicopathological information was analysed using R software (version 3.6.3) [22]. The Wilcoxon signed-rank test and Wilcoxon rank-sum test were used to compare the difference in GRAMD1A expression between KIRC tissue and normal kidney tissue, and ROC curves constructed using the pROC software package were used to evaluate the diagnostic value of GRAMD1A for KIRC [23]. Univariate and multivariate COX analyses were used to determine the effect of GRAMD1A on cancer prognosis, and Spearman correlation analysis was used to characterise the correlation between GRAMD1A expression with MSI, TMB, immune checkpoint genes, and TILs. *P* < 0.05 was considered a statistically significant difference.

## 3. Results

### 3.1. Expression of GRAMD1A in KIRC and Relationship to Pathological Features

**Figure 1:**
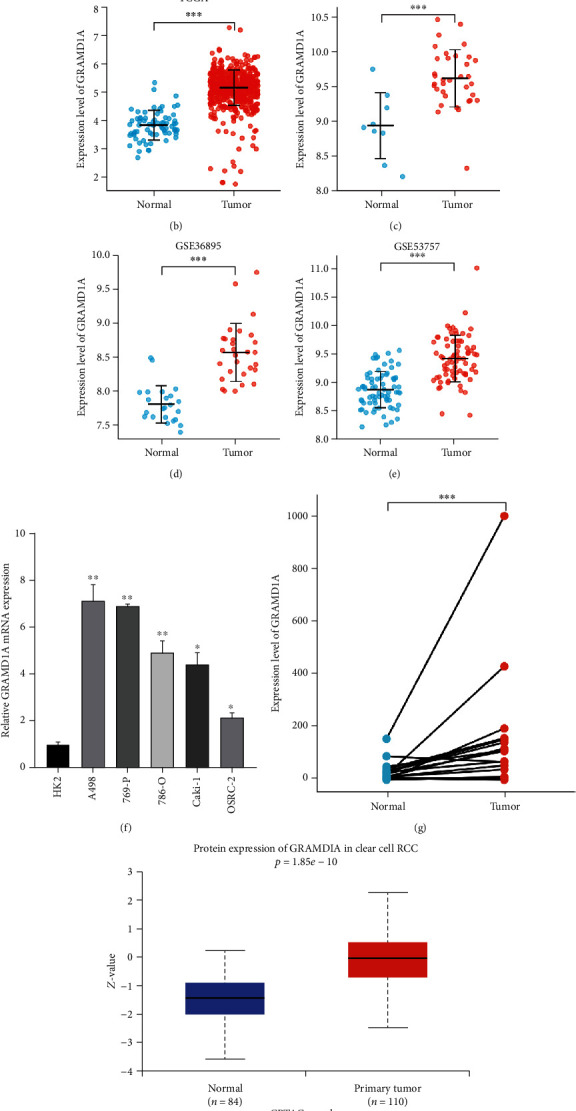
Expression of GRAMD1A in human cancer tissues. (a) Pan-cancer analysis of GRAMD1A. (b–e) Data from TCGA, GSE105261, GSE36895, and GSE53757 showed that GRAMD1A was highly expressed in KIRC samples. (f, g) GRAMD1A is highly expressed in kidney cancer cell lines as well as in KIRC tissues from patients at our medical centre. (h, i) High protein expression of GRAMD1A in KIRC tissues. ^∗^*P* < 0.05, ^∗∗^*P* < 0.01, and ^∗∗∗^*P* < 0.001. GRAMD1A: GRAM structural domain-containing protein 1A; KIRC: kidney renal clear cell carcinoma.

**Figure 2:**
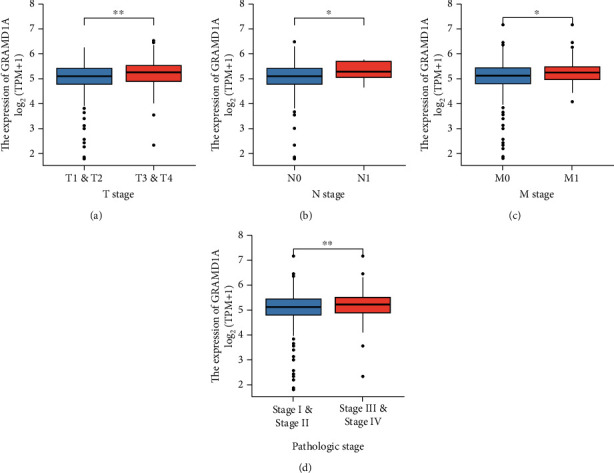
Relationship between GRAMD1A and clinicopathological characteristics of KIRC patients. (a–d) Upregulation of GRAMD1A positively correlates with TNM stage and pathological stage. ^∗^*P* < 0.05 and ^∗∗^*P* < 0.01. GRAMD1A: GRAM structural domain-containing protein 1A; KIRC: kidney renal clear cell carcinoma.

A pan-cancer analysis of GRAMD1A was performed on the Sangerbox website, and results showed that GRAMD1A was highly expressed in a variety of cancers, involving the respiratory, digestive, and urinary systems, with the greatest difference in the urinary system between KIRC and matched normal tissue (Figure 1(a)). Furthermore, we not only demonstrated differential expression of GRAMD1A in KIRC and normal tissues through multiple public datasets but also confirmed a significant upregulated state of GRAMD1A in KIRC in cell lines as well as in 19 paired tissues from our centre (Figures 1(b)–1(g)). As expected, GRAMD1A protein expression was similarly upregulated in KIRC patients and its distribution was diffused in KIRC, whereas in normal renal tissues, it was predominantly in the renal tubules (Figures 1(h) and 1(i)). To explore the potential clinical significance of GRAMD1A, we analysed its relationship with pathological characteristics and the results showed that upregulation of GRAMD1A expression was associated with higher T-stage, lymph node metastasis, distant metastasis, and high pathological stage (Figures 2(a)–2(d)).

### 3.2. Diagnostic Value of GRAMD1A

**Figure 3:**
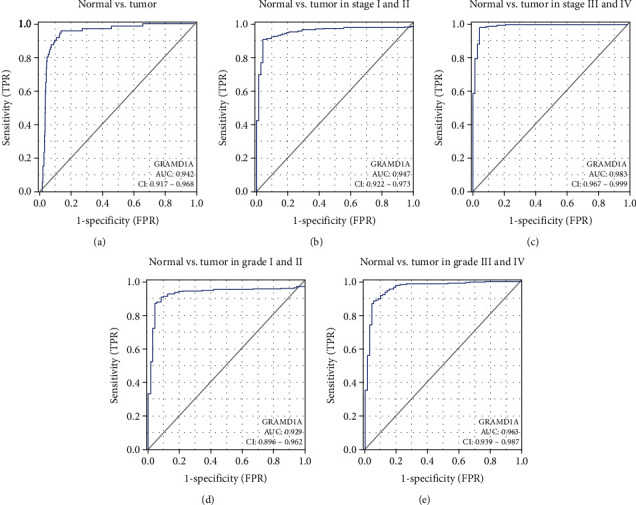
Diagnostic value analysis of GRAMD1A. (a) ROC curves of GRAMD1A expression in normal and tumour tissues, and subgroup analysis of patients with pathological stages (b) I-II and (c) III-IV and histological grades (d) I-II and (e) III-IV, respectively. GRAMD1A: GRAM structural domain-containing protein 1A.

The ROC curve was used to evaluate the diagnostic efficacy of GRAMD1A in patients with KIRC. The AUC value for differentiating normal tissue from KIRC tissue was 0.942 and for KIRC tissue at pathological stages I-II and III-IV and histological stages I-II and III-IV was 0.947, 0.983, 0.929, and 0.963, respectively (Figures 3(a)–3(e)), suggesting that GRAMD1A has a satisfactory diagnostic value for KIRC.

### 3.3. Impact of GRAMD1A Expression on the Prognosis of Human Cancers

**Figure 4:**
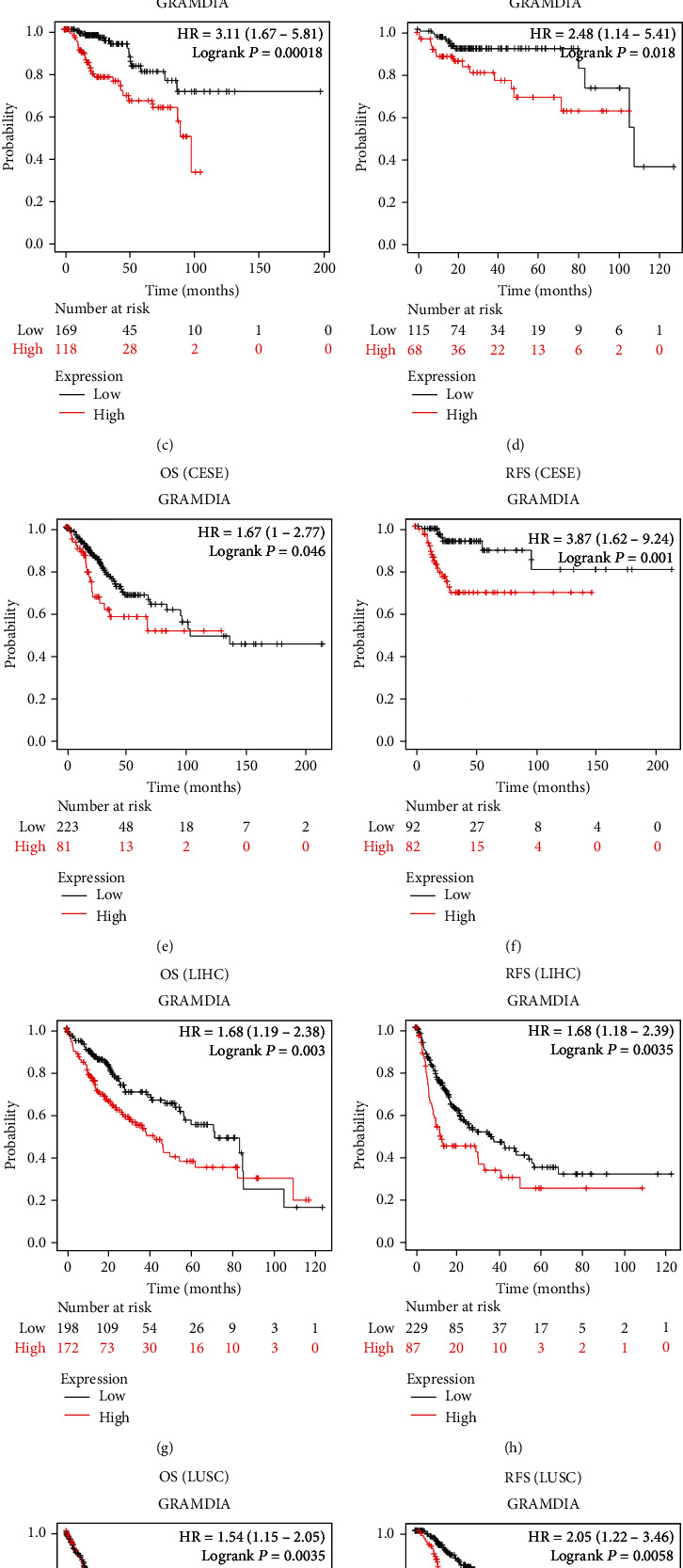
Kaplan-Meier analysis showing the relationship between GRAMD1A expression and OS as well as RFS in human cancers. (a, b) KIRC, (c, d) KIRP, (e, f) CESC, (g, h) LIHC, and (i, j) LUSC. GRAMD1A: GRAM structural domain-containing protein 1A; OS: overall survival; RFS: relapse-free survival; KIRC: kidney renal clear cell carcinoma; KIRP: kidney renal papillary cell carcinoma; CESC: cervical squamous cell carcinoma; LIHC: liver hepatocellular carcinoma; LUSC: lung squamous cell carcinoma.

**Figure 5:**
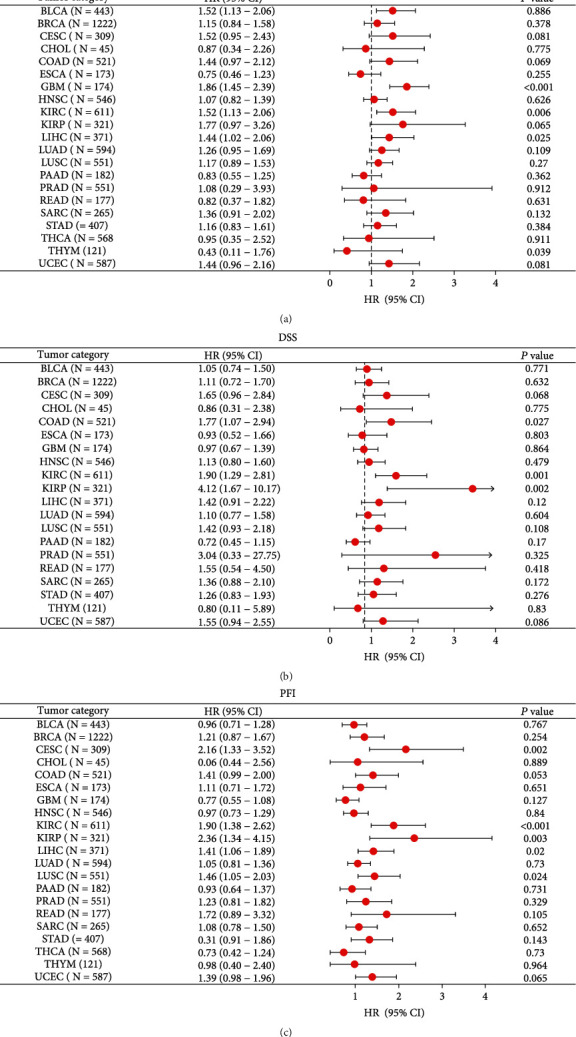
Forest plot of the prognostic value of GRAMD1A in human cancer subgroups. Prognostic HR of GRAMD1A in different cancers for (a) OS, (b) DSS, and (c) PFI. GRAMD1A: GRAM structural domain-containing protein 1A; OS: overall survival; DSS: disease-specific survival; PFI: progression-free interval.

We used the Kaplan-Meier plotter database to explore the impact of GRAMD1A on the prognosis of a variety of human cancers. High expression of GRAMD1A predicted poorer OS and relapse-free survival (RFS) for most cancers, including kidney cancer, cervical squamous cell carcinoma, liver cancer, and lung squamous carcinoma, especially in KIRC patients (Figure 4). In addition, we further validated the prognostic value of GRAMD1A in human cancers using forest plots and found a strong association between GRAMD1A and OS, DSS, and progression-free interval (PFI) in KIRC patients (Figures 5(a)–5(c)). In patients with KIRC, univariate and multivariate COX analyses assessed the impact of multiple independent factors on OS, demonstrating that age, T-stage, lymph node metastasis, distant metastasis, histological grade, and GRAMD1A expression level could be considered as independent prognostic factors for patients (Table 1). Obviously, these results suggested that high GRAMD1A expression predicted a poor prognosis in KIRC.

### 3.4. Construction and Evaluation of Prognostic Nomograms Based on GRAMD1A

**Figure 6:**
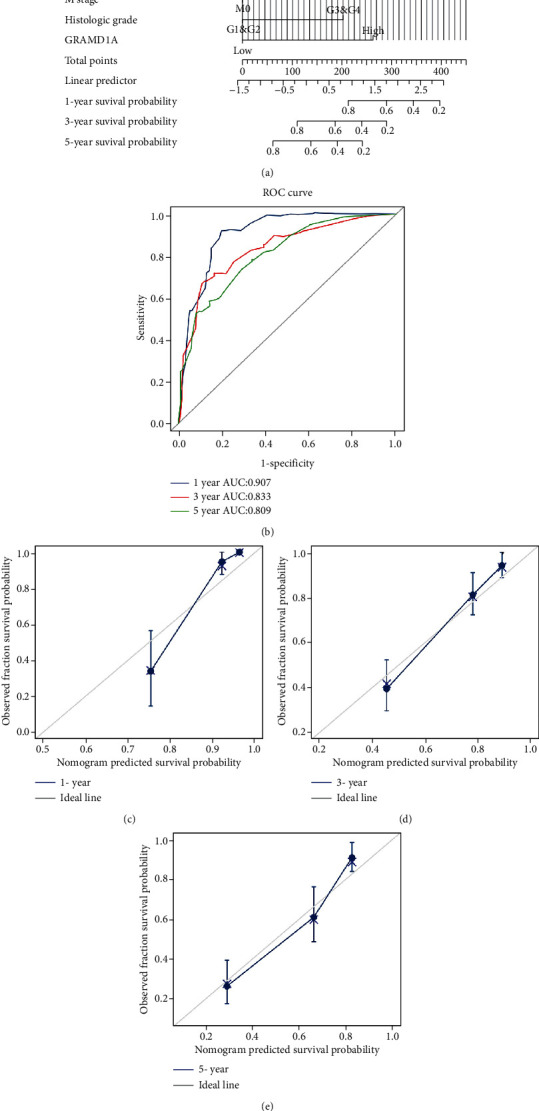
Construction and validation of the nomogram. (a) The nomogram for predicting 1-year, 3-year, and 5-year OS of KIRC patients. (b) Time-dependent ROC curves for evaluating the predictive performance of nomogram. (c–e) The calibration plots evaluating nomogram efficacy in KIRC patients at 1, 3, and 5 years for OS. KIRC: kidney renal clear cell carcinoma; OS: overall survival; DSS: disease-specific survival.

Based on the results of the COX analysis, we incorporated indicators of prognostic value for KIRC patients into the construction of nomograms to predict the probability of OS for patients at 1, 3, and 5 years, with a C-index of 0.776 (Figure 6(a)). Besides, the nomogram AUC values for OS at 1, 3, and 5 years were 0.907, 0.833, and 0.809, respectively (Figure 6(b)). At the same time, we used calibration plots to evaluate the performance of the prediction model, and the results showed good agreement between our predictions and the actual results (Figures 6(c)–6(e)).

### 3.5. Correlation of GRAMD1A Expression with MSI and TMB in Human Cancer

**Figure 7:**
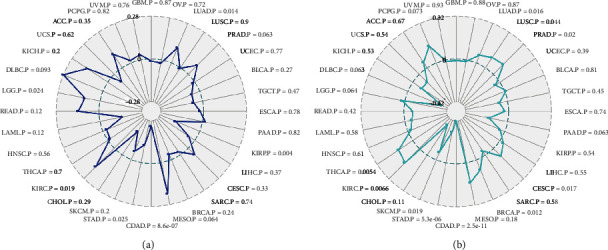
Relationship between GRAMD1A with (a) MSI and (b) TMB in KIRC. GRAMD1A: GRAM structural domain-containing protein 1A; KIRC: kidney renal clear cell carcinoma; MSI: microsatellite instability; TMB: tumour mutational burden.

Given that immunotherapy has become a first-line treatment option for a wide range of solid tumours, we have analysed the relationship between GRAMD1A with MSI and TMB in human cancer through Sangerbox website tools. Intriguingly, we found a positive association of GRAMD1A with both MSI and TMB in KIRC patients (Figures 7(a) and 7(b)).

### 3.6. Association of GRAMD1A with Immune Checkpoint Genes

**Figure 8:**
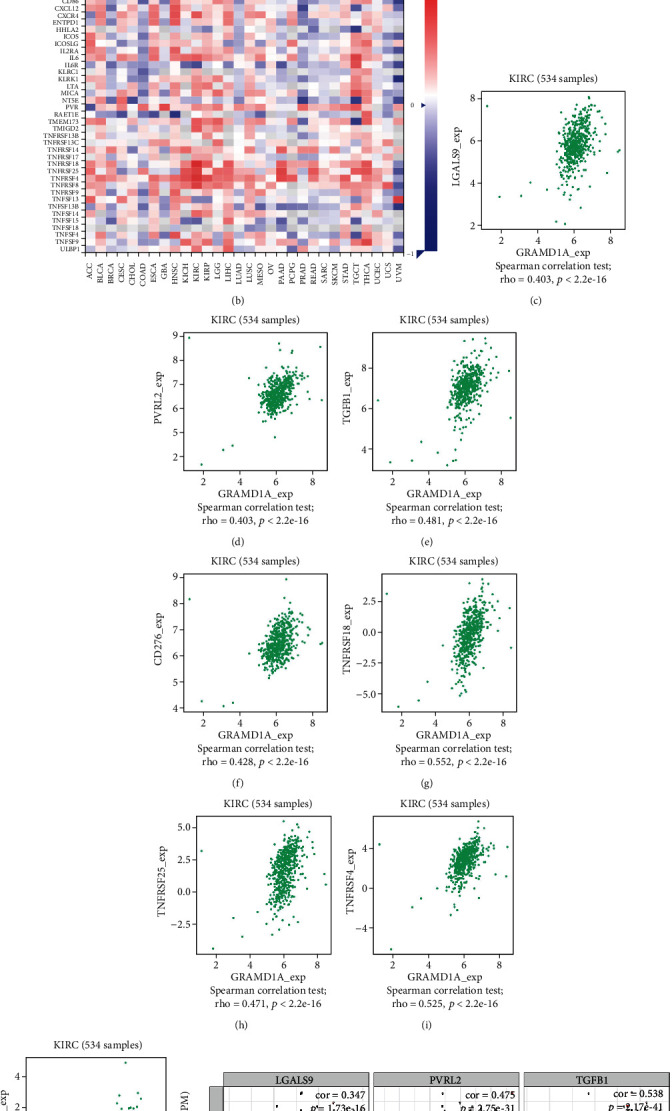
Correlation analysis of GRAMD1A expression with immune checkpoint genes based on the TISIDB and TIMER databases. (a, b) Plot of GRAMD1A expression versus immune checkpoint genes in multiple cancer types. (c–l) GRAMD1A expression was positively correlated with the expression levels of TGFB1, PVRL2, LGALS9, CD276, TNFRSF4, TNFRSF8, TNFRSF18, and TNFRSF25 based on the TISIDB and TIMER databases. GRAM structural domain-containing protein 1A.

Immune checkpoint gene blockade has been increasingly recognized as a promising treatment strategy for human cancers. With the help of the TISIDB and TIMER databases, we found strong correlations between GRAMD1A and a number of immune checkpoint genes in KIRC, including TGFB1, PVRL2, LGALS9, CD276, TNFRSF4, TNFRSF8, TNFRSF18, and TNFRSF25 (Figures 8(a)–8(j)). Furthermore, we also further validated these genes in the TIMER database for the confidence of the results (Figures 8(k) and 8(l)).

### 3.7. Relationship between GRAMD1A with TILs

**Figure 9:**
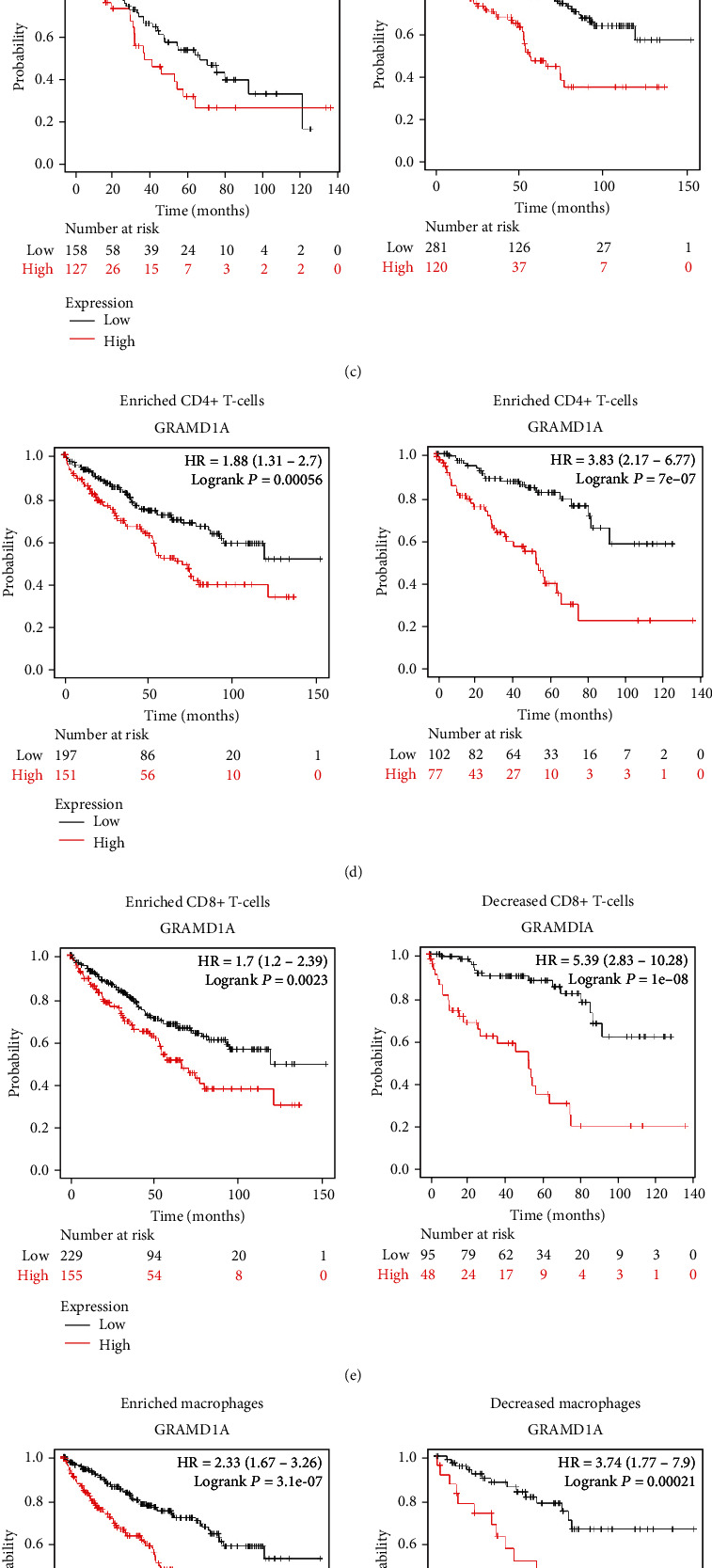
Relationship between GRAMD1A and immune infiltration in KIRC. (a) Correlation of GRAMD1A with 22 TILs in KIRC. (b–g) The effect of GRAMD1A expression on OS under conditions of enrichment or absence of major TILs in KIRC. ^∗^*P* < 0.05. GRAMD1A: GRAM structural domain-containing protein 1A. KIRC: kidney renal clear cell carcinoma. TILs: tumour-infiltrating lymphocytes.

Based on the CIBERSORT algorithm, we analysed the correlation of GRAMD1A with 22 TILs in KIRC and showed that GRAMD1A was positively correlated with neutrophils, naive B cells, and plasma cells and negatively correlated with gamma delta T cells, and eosinophilic (Figure 9(a)). To further analyse the relationship between GRAMD1A with the immunity, we used the Kaplan-Meier plotter to compare the effect of GRAMD1A on OS in KIRC patients in enriched and deficient states of immune-infiltrating lymphocytes in the tumour microenvironment (TME). Notably, the hazard ratio (HR) of death in KIRC patients with high GRAMD1A expression was relatively smaller when B lymphocytes, natural killer T cells, CD4+ T lymphocytes, CD8+ T lymphocytes, and macrophages were enriched in the TME (Figures 9(b)–9(f)), whereas the HR of death in such patients was greater when regulatory T lymphocytes were significantly enriched (Figure 9(g)).

### 3.8. Potential Signalling Pathways of GRAMD1A

**Figure 10:**
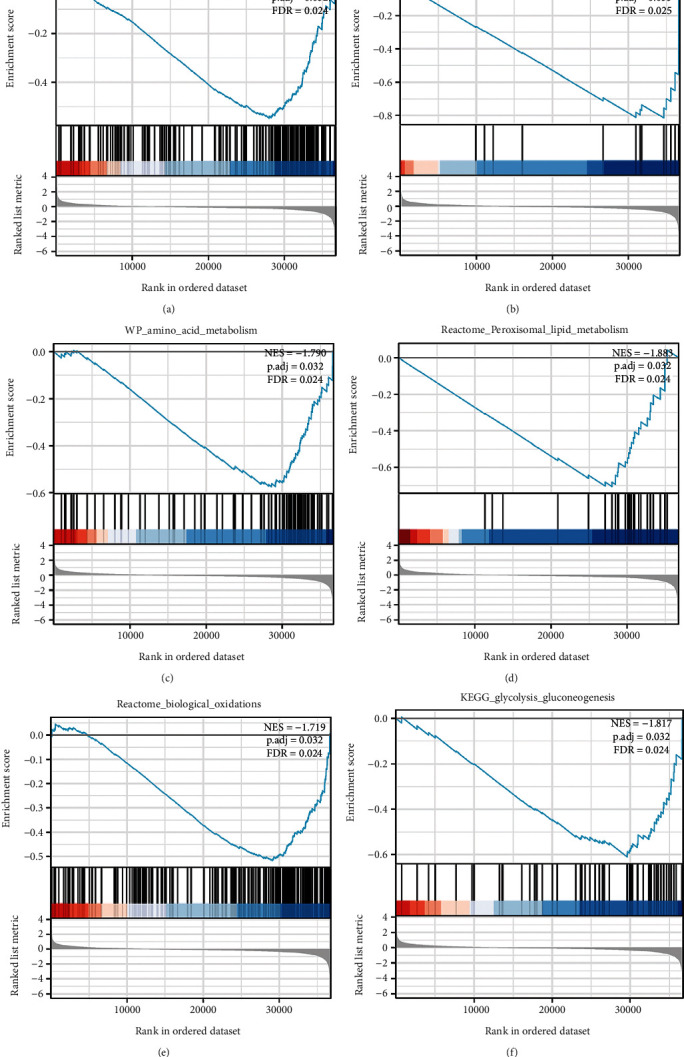
Enrichment plots from GSEA: (a) Fatty acid metabolism, (b) fatty acid omega oxidation, (c) amino acid metabolism, (d) peroxisomal lipid metabolism, (e) biological oxidation, and (f) glycolysis gluconeogenesis. GSEA: gene set enrichment analysis.

To explore the biological mechanisms of GRAMD1A in KIRC, we used GSEA to analyse its potential signalling pathways. As shown in Figure 10, fatty acid metabolism, fatty acid omega oxidation, amino acid metabolism, peroxisomal lipid metabolism, biological oxidation, and glycolysis gluconeogenesis can be significantly enriched. Based on these findings, we speculate that the role of GRAMD1A in KIRC may be closely related to energy metabolism.

## 4. Discussion

GRAMD1A is widely expressed in the nucleus and cytoplasm of embryonic stem cells, cancer cell lines, and ectodermal, mesodermal, and endodermal tissues, and its function has not been fully explored [8]. In previous studies, GRAMD1A was found to accumulate at autophagosome initiation sites, influence cholesterol distribution during starvation, and participate in autophagosome biogenesis [24]. Besides, as one of the genes in the TLR7 regulatory pathway, GRAMD1A can be used to assess the response of psoriasis after treatment [25]. In human cancers, GRAMD1A is currently only found to promote proliferation, migration, and invasion of hepatocellular carcinoma as well as the expansion of hepatocellular carcinoma stem cells [10, 11]. In the present study, we firstly found that GRAMD1A was highly expressed in a variety of cancer tissues by pan-cancer analysis, including kidney, breast, colon, bile duct, and lung cancers, among others. Subsequently, the upregulation of GRAMD1A in KIRC was also validated in a number of renal cancer cell lines and in KIRC tissues from patients at our medical centre. In addition, we found that GRAMD1A expression positively correlated with clinicopathological stage and histological grade, suggesting that GRAMD1A may have potential as a prognostic biomarker for KIRC patients.

To explore the clinical value of GRAMD1A, ROC curves were used to assess its diagnostic efficacy for KIRC, and it was found that GRAMD1A had satisfactory AUC values for differentiating various types of KIRC from normal kidney tissue. We also assessed the impact of GRAMD1A on the prognosis of human cancers by Kaplan-Meier analysis and forest plots, and for most cancers, upregulation of GRAMD1A expression was an unfavourable prognostic factor, a feature that was most prominent in KIRC patients. Subsequently, univariate and multivariate COX analyses further analysed the factors that may affect OS in patients with KIRC, and the results suggested that age, TNM stage, histological grade, and GRAMD1A expression could be independent prognostic factors for KIRC patients. Based on these systematic analyses, it is reasonable to conclude that GRAMD1A could be a valid diagnostic and prognostic biomarker for KIRC. In combination with the valuable prognostic indicators derived from the COX analysis, we further constructed nomograms to predict the probability of OS for KIRC patients at 1, 3, and 5 years. Meanwhile, the C-index, time-dependent ROC curve, and calibration curves confirmed their moderate accuracy and clinical applicability.

KIRC has long been considered chemoresistant and still fails to respond well to targeted antiangiogenic therapies and immune checkpoint inhibitors in the majority of patients [26, 27]. Moreover, GRAMD1A has been found to be involved in possible resistance to chemotherapy in patients with hepatocellular carcinoma [10]. However, whether GRAMD1A can influence resistance to immunotherapy remains uncovered, making it a proposition worth exploring whether GRAMD1A can be involved in the regulation of the immune microenvironment and immune checkpoints in KIRC. MSI and TMB are considered to be reliable biomarkers for immune checkpoint inhibitors and prognosis of cancer patients [28–30]. In this study, we used radar plots to clearly demonstrate the association of GRAMDIA with MSI and TMB in human cancers, and as expected, GRAMD1A was positively correlated with both MSI and TMB in KIRC. Furthermore, we analysed the association of GRAMD1A with immune checkpoint genes and the molecules with strong correlation included TGFB1, PVRL2, LGALS9, CD276, TNFRSF4, TNFRSF8, TNFRSF18, and TNFRSF25. Of note, in previous studies, TGFB1 has been found to enhance proliferative and metastatic potential by upregulating lymphoid enhancer-binding factor 1 and integrin *α*M*β*2 in human renal cell carcinoma [31], and PVRL2 has been found to be induced in cancer and to suppress CD8+ T lymphocyte function [32]. In parallel, CD276 is overexpressed in tumour tissue and is involved in the shaping and development of TME [33], while members of the TNF family are thought to regulate cell differentiation, survival, and programmed death and are associated with the immune system [34]. These results suggest that GRAMD1A is associated with immune infiltration in TME and may be partially involved in the immunoregulatory processes of KIRC.

KIRC is highly immunoinfiltrative, with infiltration of TILs in its tumour microenvironment occupying a key position and being closely associated with the patient's response to immunotherapy and prognosis [35, 36]. Endogenous inflammation in cancer cells can expand during KIRC progression, leading to neutrophil-dependent distant metastases [37]. In contrast, the gamma delta T cells have been found to recognize certain RCC-associated antigens and play an active role in the surveillance system for RCC [38]. Interestingly, our results suggest that GRAMD1A correlates with increased abundance of neutrophils, while negatively correlating with the abundance of gamma delta T cells. Clearly, this is consistent with a malignant phenotype of GRAMD1A in KIRC. Furthermore, we analysed the impact of GRAMD1A on the survival of KIRC patients in a state of TILs enrichment or deficiency. When B lymphocytes, natural killer T cells, CD4+ T lymphocytes, and CD8+ T lymphocytes were enriched, patients with high GRAMD1A expression had a lower HR of death compared to when these lymphocytes were deficient. This phenomenon may be explained in part by the fact that these lymphocytes produce suppression of tumour growth during immune regulation [39–41], since, on the contrary, patients with high GRAMD1A expression have a higher HR of death when enriched in regulatory T cells that possess tumour-specific immunosuppressive effects [42]. Therefore, considering the specific function of these lymphocytes in TME and these representative results, it seems reasonable to infer that TILs are involved to some extent in the detrimental effects of GRAMD1A in patients with KIRC.

In the meantime, KIRC is characterised by reprogramming of energy metabolism, mainly including glucose metabolism, lipid metabolism, and impaired mitochondrial bioenergetics and oxidative enzymes [43–47]. In particular, a recent study has mapped the lipidome profile of human KIRC and combined this with transcriptomic data, which has undoubtedly deepened the understanding of the TME of KIRC [48]. Besides, evidence suggests that GRAMD1A may be involved in regulating cholesterol metabolism, transport, and autophagosome biogenesis [24, 49]. However, it is encouraging that our GSEA results predict that GRAMD1A may be involved in processes such as gluconeogenesis, fatty acid metabolism, amino acid metabolism, and biological oxidation in KIRC. Thus, supported by this evidence, how GRAMD1A is involved in the regulation of energy metabolism in KIRC deserves to be explored in depth in the next study.

Despite the systematic analysis of GRAMD1A in this study, there are still some shortcomings that should not be overlooked. Firstly, the clinicopathological information of the KIRC patients in this study was obtained from public databases and the relevant treatment information is incomplete. Second, the functional and mechanistic study of GRAMD1A in KIRC in this paper is bioinformatics-based and lacks further experimental validation in vivo and in vitro.

## 5. Conclusion

Here, this study revealed through data analysis that GRAMD1A expression was upregulated in KIRC tissue and was strongly associated with poor prognosis of patients. The ROC curve and multivariate COX analysis further clarified the good diagnostic and prognostic value of GRAMD1A in KIRC. Furthermore, we found that GRAMD1A correlated with immune checkpoint genes, MSI and TMB, and that the adverse effects of GRAMD1A on KIRC patients were to some extent dependent on the immunomodulatory effects of TILs.

## Figures and Tables

**Table 1:** Cox regression analysis of GRAMD1A expression level and clinicopathological features in KIRC.

Characteristics	Total (*N*)	Univariate analysis		Multivariate analysis	
		Hazard ratio (95% CI)	*P* value	Hazard ratio (95% CI)	*P* value
Gender	539				
Female	186	Reference			
Male	353	0.930 (0.682-1.268)	0.648		
Age	539				
≤60	269	Reference			
>60	270	1.765 (1.298-2.398)	<0.001	1.755 (1.138-2.706)	0.011
Laterality	538				
Left	252	Reference			
Right	286	0.706 (0.523-0.952)	0.023	1.025 (0.663-1.585)	0.910
T stage	539				
T1&T2	349	Reference			
T3&T4	190	3.228 (2.382-4.374)	<0.001	1.890 (1.182-3.021)	0.008
N stage	257				
N0	241	Reference			
N1	16	3.453 (1.832-6.508)	<0.001	1.866 (0.937-3.714)	0.076
M stage	506				
M0	428	Reference			
M1	78	4.389 (3.212-5.999)	<0.001	2.963 (1.839-4.776)	<0.001
Histologic grade	531				
G1&G2	249	Reference			
G3&G4	282	2.702 (1.918-3.807)	<0.001	1.643 (0.993-2.719)	0.053
GRAMD1A	539				
Low	270	Reference			
High	269	1.543 (1.141-2.087)	0.005	1.904 (1.238-2.929)	0.003

## Data Availability

Both RNA-seq and clinicopathological data for KIRC patients are available from the database (https://portal.gdc.cancer.gov/) and the GEO database (https://www.ncbi.nlm.nih.gov/gds/); further inquiries can be directed to the corresponding authors.
